# Predictors of the worry about cancer recurrence among women with breast cancer

**DOI:** 10.1186/s12905-023-02296-1

**Published:** 2023-03-25

**Authors:** Masoumeh Safdari-Molan, Esmat Mehrabi, Roghaiyeh Nourizadeh, Reza Eghdam-Zamiri

**Affiliations:** 1 Midwifery Department, Nursing and Midwifery faculty, Tabriz University of Medical Sciences, Tabriz, Iran; 2 Medical School, Tabriz University of Medical Sciences, Tabriz, Iran

**Keywords:** Breast cancer, Chemotherapy, Worry about the cancer recurrence, Radiotherapy, Fear of cancer

## Abstract

**Introduction:**

Worry about cancer recurrence is identified as the most common psychological burdens experienced by cancer patients and survivors. The present study aimed to determine the predictors of worry about cancer recurrence among women with breast cancer.

**Materials and methods:**

This cross-sectional study was conducted on 166 women with breast cancer undergoing chemotherapy and radiotherapy, who referred to private and public oncology centers in Tabriz, Iran using the convenience sampling. Data collection tools were demographic and disease characteristics questionnaire, cancer worry scale, social support questionnaire, brief illness perception questionnaire, international physical activity questionnaire-short form, and The EORTC-in-patsat32. The data were analyzed using SPSS 25 software. Pearson correlation coefficient, independent t-test, ANOVA, and multivariate linear regression were used.

**Results:**

In the present study, the mean (standard deviation) of score of worry about cancer recurrence was 17.41 (7.88), ranging from 8–32. The results revealed that the type of surgery, illness perception, satisfaction with care, and place of treatment were the most important predictors of worry about cancer recurrence, which explained 44.3% of the variance.

**Conclusion:**

The enhancement of satisfaction with care and training coping strategies among individuals with high perceived severity of the illness contribute to the reduction of worry about cancer recurrence and adaptation to breast cancer.

## Introduction

Female breast cancer is considered as the most common cancer, with an estimated 2.3 million new cases (11.7%) [1]. Further, breast cancer is very common among Iranian women, which represents 24.6% of all cancers. The average annual crude incidence of primary breast cancer is 22.6 per 100,000 Iranian women [2]. The incidence of breast cancer in Iran has increased significantly in the last two decades, due to the change in lifestyle and reproductive behaviors and its trend is expected to increase in the future, because of the correlation with population aging [3, 4]. About 80% of all breast cancers arise in women over age 50; and the 10-year probability of developing invasive breast cancer increases from less than 1.5% at age 40, to about 3% at age 50 and over 4% by age 70, producing a cumulative lifetime risk of 13.2% or 1 in 8 [5].

Improving the cancer diagnosis and treatment methods has led to an increase in the number of cancer survivors for a long time. One of the biggest worries of breast cancer survivors is about cancer recurrence, which women with breast cancer experience its varying degree in their survival path [6–11]. Worry about cancer recurrence is recognized as the most common psychological burdens experienced by cancer patients and survivors. Based on the results of previous studies, 39–97% of cancer survivors experience worry about cancer recurrence and its manifestation range varies from fear as a natural response to cancer to behavioral disorders, depression, and distress [9]. High severity of worry about cancer recurrence is significantly associated with lower quality of life, functional disorders, and increased health care costs [12].

In addition, several early studies conducted on cancer survivors, such as breast, gynecologic, colorectal, and head and neck cancers, demonstrated that the high severity of worry about recurrence were more correlated with demographic, medical, and psychological characteristics, such as cancer diagnosed at young age, low elapsed time from the diagnosis of cancer, more advanced disease, low level of satisfaction with care, low social support, illness perception, high pain, and disability. However, these findings are sometimes contradictory in different studies [13–16].

More knowledge about predictors of worry about cancer recurrence among survivors may help health care providers in designing interventions and improving patients’s quality of life [12]. Therefore, considering the limited number of studies focused on the worry about cancer recurrence among breast cancer survivors, the present study aimed to determine the predictors of worry about cancer recurrence among women with breast cancer.

## Methods

### Procedure

This cross-sectional study was conducted on women with breast cancer who referred to public and private oncology centers in Tabriz, Iran for chemotherapy or radiotherapy from October 2021 to February 2022. The inclusion criteria were getting primary breast cancer based on the medical record with each stage, more than one month elapsed from the diagnosis, and undergoing chemotherapy or radiotherapy. The exclusion criteria included having a history of chronic and systemic diseases, having a history of mental disorders, having other concurrent cancers or breast cancer metastases based on the medical record, undergoing chemotherapy or radiotherapy due to the cancer recurrence, and experiencing other stressful events, such as death of relatives during the last six months.

The sample size was calculated using the formula $\mathrm{n}=\frac{{\mathrm{Z}}_{1-\frac{\mathrm{\alpha }}{2}}^{2} {\delta }^{2}}{{\mathrm{d}}^{2}}$ [17] and based on the study of Konings et al. [18]. Thus, considering SD = 3.4, d = 0.05 around the mean (14.4), α = 0.05 with 95% confidence coefficient, and 20% sample drop, the sample size was obtained 166 subjects. The sampling was done using the convenience sampling after obtaining the permission of the Ethics Committee of Tabriz University of Medical Sciences (IR.TBZMED.REC.1398.577). Convenience sampling is a non-probability sampling that involves the sample being drawn from that part of the population that is close to hand [19]. In the same vein, the researcher referred to the public and private oncology centers in Tabriz, evaluated the patients in terms of the inclusion and exclusion criteria, provided the information about the research objectives and confidentiality of information, and asked the eligible patients to participate in the study. The written informed consent was obtained from all participants. The eligible women who were reluctant to participate in the study, were not included.

Therefore, 166 women with breast cancer undergoing chemotherapy and radiotherapy (83 subjects from public centers and 83 from private centers) completed the demographic and disease characteristics questionnaire, cancer worry scale, social support questionnaire, brief illness perception questionnaire, international physical activity questionnaire-short form, and the EORTC-in-patsat32 through interview in a quiet environment. Some important questions, such as the type and stage of cancer were completed from the patients’ files.

### Measures

The demographic and disease characteristics questionnaire included the variables of age, educational level, occupation, family income level, insurance coverage, place of residence, marital status, number of children, type and stage of breast cancer, place of treatment, and type of surgery as well as elapsed time from the diagnosis of cancer.

The present study used the eight-item cancer worry scale (CWS). According to Custers et al. [20], the CWS is a reliable and valid questionnaire to assess fear of recurrence in breast cancer survivors. It can screen and assist survivors in accessing support. The responses are rated on a 4-point Likert scale ranging from 1: never to 4: almost always. The scores are ranged from 8 – 32. Higher scores represent more worry about cancer recurrence. The Cronbach’s alpha of the scale was 0.88 [21]. The reliability of the scale in Iran was confirmed by Safdari et al. Internal consistency of the questionnaire was 0.81 [22].

The brief illness perception questionnaire (Brief IPQ) with nine items was used to assess the illness perception of women with breast cancer. The first eight items are scored on a scale of 1–10. The item nine is open-ended, questioning the three main causes of the illness. The scores are ranged from 0–80. The Cronbach’s alpha coefficient of the tool was reported to be 0.80 and the test–retest reliability was 0.75 [23]. Karimi et al. [24] confirmed the validity of the questionnaire in Iran.

Further, the EORTC-in-patsat32 questionnaire, developed by Bredart et al. [25], was applied to measure the patient’s satisfaction with medical care. The questionnaire evaluates the areas of satisfaction with health care providers, and aspects of the organization of care and services. The responses are rated on a 5-point Likert scale (1: poor; 2: average; 3: good; 4: very good; 5: excellent) and the score of each area is between 0–100 and higher score represents more satisfaction. The Cronbach’s alpha of the tool was calculated to be 0.56—0.96. In the Persian version of the EORTC In-Patsat32 [26], internal consistency for all domains was high (α > 0.90) and the test–retest reliability was excellent (r = 0.86–0.96).

The present study applied the twelve-item social support questionnaire developed by zimet et al. [27]. The multidimensional scale of perceived social support (MSPSS) is a 7-point Likert scale ranging from 1: strongly disagree to 7: strongly agree. The total score ranges from 12–84 and the higher the score, the greater the perceived social support. The Cronbach’s alpha of the scale and Cronbach's α coefficient were reported as 0.83 and 0.84, respectively. The test–retest reliability was yielded 0.84 for the Iranian version of the MSPSS [28].

In addition, the present study used the international physical activity questionnaire—short form (IPAQ-SF) with seven items during the last seven days. The physical activity level is classified into the light physical activity, like walking, moderate physical activity, such as carrying light loads, cycling at average speed, and playing volleyball, and heavy physical activity, such as lifting heavy objects, digging, like digging a garden, aerobic exercise, fast cycling, and running. For Calculating and classifying the physical activity, the metabolic equivalents (MTEs) were calculated for the aforementioned physical activities. The metabolic equivalent is considered to be 3.3 for walking, 4 for moderate activity, and 8 for vigorous activity. Then, the numbers were multiplied by the duration of performed activity (min) and the number of the days of doing that activity [29]:

$$\mathrm{Metabolic}\;\mathrm{equivalent}\;(\min\;\mathrm{per}\;\mathrm{week})=\mathrm{walking}\;(\mathrm{metabolic}\;\mathrm{equivalent}\times\min\times\mathrm{day})+\mathrm{moderate}\;\mathrm{activity}\;(\mathrm{metabolic}\;\mathrm{equivalent}\times\min\times\mathrm{day})+\mathrm{vigorous}\;\mathrm{activity}\;(\mathrm{metabolic}\;\mathrm{equivalent}\times\min\times\mathrm{day})$$

The Cronbach’s coefficient of 0.7 was demonstrated for the Persian version of IPAQ-SF [30].

### Statistical analysis

The data were analyzed using SPSS / Ver 25 software and Kolmogorov–Smirnov test was used to measure the data distributions for normality. The data were analyzed using independent t-test, Pearson correlation coefficient, and ANOVA. Then, the independent variables with *P*-value of less than 0.2 in the bivariate test entered into the multivariate linear regression model through backward strategy in order to control the confounding variables.

## Results

The mean age of patients was 51.28 (11.85) years and most of them (92.8%) had insurance. Further, 44.5% and 55.4% patients were treated with radiotherapy and chemotherapy, respectively. About half of the subjects (55.4%) underwent lumpectomy. The most common type of illness was carcinoma in situ (62.0%). Of the 37.9% with invasive cancer, the commonest were those with stage one (46.9%) (Tables 1 and 2).

**Table 1:** The demographic characteristics of the participants and its relationship with the worry about cancer recurrence (*n* = 166)

Demographic Characteristics	No. (%)	Worry of Cancer M(SD)	*P*-value
**Age/mean(SD)**	51.28(11.85)	17.41(7.88)	*r* = -114, 0.145 ^a^
**Number of children/mean(SD)**	3.12(1.39)	17.41(7.88)	*r* = 0.038, 0.629 ^a^
**Husband`s age/mean(SD)**	57.75(11.14)	17.41(7.88)	*r* = -0.136, 0.129 ^a^
**Insurance**			t = -0.038, 0.969 ^b^
Yes	154(92.8)	17.40(8.08)	
No	12(7.2)	17.50(4.73)	
**Job**			F = 0.370, 0.774 ^c^
Housewife	142(85.5)	17.52(7.98)	
Employee	7(4.2)	19.14(7.88)	
Retired	12(7.2)	15.50(6.80)	
Others	5(3.0)	16.60(8.76)	
**Education**			F = 1.226, 0.302 ^c^
Illiterate	68(40.9)	16.01(7.66)	
Elementary	42(25.3)	18.52(8.70)	
High school	37(22.2)	18.29(7.57)	
Academic	19(11.4)	18.26(7.12)	
**Husband`s education**			F = 1.655, 0.180 ^c^
Illiterate	44(26.5)	15.92(7.32)	
Elementary/Guidance	56(33.7)	17.57(8.98)	
High school/Diploma	39(23.4)	18.37(6.49)	
Academic	17(10.2)	20.23(7.52)	
**Income**			F = 2.412, 0.093 ^c^
Sufficient	21(12.6)	19.47(8.56)	
Relatively sufficient	59(35.5)	18.50(7.53)	
Insufficient	86(51.8)	16.16(7.81)	
**Marital status**			F = 1.003, 0.393 ^c^
Married	124(74.6)	17.54(7.58)	
Unmarried	20(12)	17.90(8.94)	
Divorced	2(1.2)	8(0.00)	
Widow	20(12)	17.05(8.75)	
**Address**			t = -0.267, 0.790 ^b^
Town	124(74.6)	17.31(7.64)	
Village	42(25.3)	17.71(8.62)	

^a^ Pearson Correlation/, ^b^ Independent t test/, ^c^ ANOVA

**Table 2:** The disease characteristics of the participants and its relationship with the worry about cancer recurrence (*n* = 166)

**Characteristics of Disease**	**No. (%)**	**worry of Cancer Recurrence** **Mean (SD)**	***p*-value**
**Type of breast disease**			t = -0.620, 0.547^a^
Carcinoma in situ	103(62.0)	17.34(8.01)	
Invasive	63(37.9)	18.50(5.52)	
**Stage of cancer**			
Stage 1	78(46.9)	15.84(7.63)	F = 2.992, 0.053^b^
Stage 2	70(42.1)	18.84(8.10)	
Stage 3	18(10.8)	18.66(7.14)	
**How to know about the disease**			t = 0.576, 0.537^a^
Directly	139(83.7)	17.58(7.75)	
Indirectly	27(16.2)	16.55(8.61)	
**Type of surgery**			t = -1.787, 0.078^a^
Mastectomy	74(44.5)	19.64(8.06)	
Lumpectomy	92(55.4)	16.66(7.04)	
**Past time of diagnosis (month)/ mean (SD)**	-	17.41(7.88)	r = 0.60, 0.442^c^
**Past time of surgery (month)/mean (SD)**	-	17.41(7.88)	**r = 0.154, 0.048**^**c**^
**Treatment center**			
Private	74(44.5)	20.12(7.15)	**t = 4.198, < 0.001**^**a**^
Public	92(55.42)	15.23(7.80)	
**Type of treatment**			t = -2.447, 0.170^a^
Chemotherapy	92(55.4)	20.02(7.42)	
Radiotherapy	74(44.5)	18.43(8.05)	

^a^ Independent t test /, ^b^ ANOVA /, ^c^ Pearson Correlation

In the present study, the mean (SD) score of worry about cancer recurrence was 17.41 (7.88) from the achievable range of 8–32. The bivariate tests indicated a significant relationship between the worry about cancer recurrence and the elapsed time from the surgery (*P* = 0.048), place of treatment (*P* < 0.001), and illness perception (*P* < 0.001) (Tables 2 and 3). After entering variables with *P* < 0.2 in the multivariate linear regression model, the predictors of worry about cancer recurrence included illness perception, satisfaction with care, type of surgery, and place of treatment, which generally explained 44.3% of the variance (Table 4).

**Table 3:** The relation of the worry of cancer recurrence with illness perception, social support, care satisfaction, and physical activity

Worry of cancer	Illness perception	Social support	Care satisfaction	Physical activity
r^a^	0.418	0.009	-0.060	-0.064
*P*-value#	** < 0.001**	0.912	0.141	0.409

^a^Pearson correlation coefficient; #Correlation is significant at the .05 level (2-tailed)

**Table 4:** Predictors of worry of cancer recurrence in women with breast cancer (*n* = 166)

Variable	β (CI 95%)^a^	*P*#
**Illness perception**	0.150 (0.077to 0.222)	**0.001**
**Place of Treatment (reference: Public)** Private	4.22 (0.516 to 7.929)	**0.026**
**Care satisfaction**	-0.051(-0.096 to -0.006)	**0.025**
**Type of surgery (reference: Lumpectomy)**		
Mastectomy	3.507(0.416 to 7.431)	**0.049**
_**Adjusted R**_^**2**^	**0.443**	

^a^Confidence interval 95%; # Multivariate Linear Regression

## Discussion

The present study aimed to determine the predictors of worry about cancer recurrence among women with breast cancer undergoing chemotherapy and radiotherapy. The most common type of illness was carcinoma in situ (62.0%). Of the 37.9% with invasive cancer, the commonest were those with stage one (46.9%). Given that most of the cancer patients in the northwest of Iran refer to Tabriz or Tehran (the capital) and even some of the patients from Tabriz also refer to Tehran, the rates reported in this study do not reflect the actual prevalence in the society.

The mean (SD) of worry about cancer recurrence score was 17.41 (7.88), ranging from 8–32. In addition, some factors, such as the type of surgery, place of treatment, satisfaction with care, and perception of the illness were considered as the predictors of the worry about recurrence among women with breast cancer.

The results indicated a significant relationship between worry about cancer recurrence and illness perception. In patients with high illness perception, the worry of recurrence was also higher. Other studies have also reported the same findings. In a study conducted in New Zealand [31], on postmenopausal women with breast cancer undergoing chemotherapy, was reported a significant correlation between the worry of cancer recurrence and all domains of illness perception except personal control. Shim et al. [32] in an investigation among women with breast cancer in Korea reported that illness perception is a determinant of worry of cancer recurrence, and noted self-efficacy and depression as the moderators of the relationship between the illness perception and the worry of cancer recurrence. In another study on Japanese and Scandinavian women [33], the illness perception was significantly associated with the worry about cancer recurrence. Further, the consistent results were reported in the study of Freeman et al. [34].

In the present study, the type of surgery was significantly associated with worry about cancer recurrence, as patients who underwent mastectomy were more worried about cancer recurrence. Since the patients underwent mastectomy due to the diagnosis of breast cancer in more advanced stages, or in some cases, they insisted on mastectomy instead of lumpectomy due to the high illness perception, worry about cancer recurrence was more in this group. Previous two studies also reported the same findings [35, 36]. However, Vickberg et al. [37] and Janz et al. [38] indicated that women who underwent lumpectomy were more worried about cancer recurrence than those who underwent mastectomy. Moreover, no difference was reported in the level of worry about cancer recurrence among patients who underwent mastectomy or lumpectomy in other studies [9, 39], the difference in the results of these studies may be due to the demographic and socio-cultural differences in the illness perception.

In the present study, a significant relationship was observed between the worry about cancer recurrence and satisfaction with care, as higher levels of satisfaction with care were associated with lower worry about cancer recurrence. In a study conducted on the patients with prostate cancer, a significant relationship was observed between worry of cancer recurrence and satisfaction with care. Since higher level of care satisfaction reduce the negative impact of the worry of cancer recurrence on patients’ quality of life [40], which is consistent with the present study.

In the present study, the place of treatment was regarded as another predictor of worry about cancer recurrence, as women who referred to private clinics had higher level of worry about cancer recurrence, which could possibly be due to their high level of education and socioeconomic status. On the other hand, the difference in providing training and support programs in private and public centers can be another reason for the difference in the illness perception and consequently, the difference in the worry about cancer recurrence among those who refer to these centers [41].

## Limitations

The research design is one of the limitations of this study. Given that this study was cross-sectional, it does not necessarily indicate a causal relationship between the variables.

## Conclusion

The enhancement of satisfaction with care and training coping strategies among individuals with high perceived severity of the illness seem to contribute to the reduction of the worry about cancer recurrence and adaptation to breast cancer.

## Data Availability

The datasets analyzed during the current study available from the corresponding author on reasonable request.
